# Synthesis and Antitumor Activity of Substituted Succinamides Using a Potato Disc Tumor Induction Assay

**Published:** 2007-03

**Authors:** Farzin Hadizadeh, Alireza Moradi, Goli Naghibi, Mojgan Vojdani, Javad Behravan, Mohammad Ramezani

**Affiliations:** 1 *Biotechnology & Pharmaceutical Research Center, Mashhad University of Medical Sciences, Mashhad, Iran;*; 2 *Pharmacy Faculty, Mashhad Univesrsity of Medical Sciences, Mashhad, Iran*

**Keywords:** succinamides, antitumor, potato disc

## Abstract

In view of potential biological activities of some succinic acid derivatives, we synthesized some novel N-[4-(4-morpholinosulfonyl)-phenyl]-succinamides (6a, c; 7a, c) and N-[4-(benzylaminosulfonyl) phenyl]-succinamides (6b, d; 7b, d) derivatives as antitumor agents. The antitumor activity of compounds was studied using the potato disk bioassay technique. Vincristine at 0.25 mg/ml was employed as positive control and caused -67.24% inhibitions. Compound 7b at 1 mg/ml caused -80.50% tumor inhibitions with highest activity among compounds tested.

## INTRODUCTION

**Figure 1:**
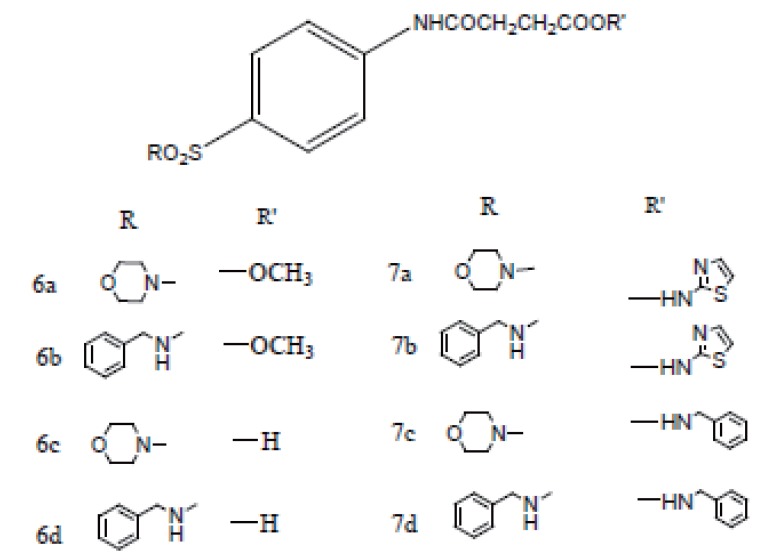
Chemical structures of succinamides **6a-d** and **7a-d**.

Previously dual antitumor and antiinflammatory activity of a series of substituted succinamic acids have been reported (1-4) Synthesis and biological activity of substituted amides and hydrazides of 1, 4-dicarboxylic acids have been recently reviewed (5). In an attempt to find novel succinamides as antitumors, we have synthesized some novel N-[4-(morpholin-4-ylsulfonyl) phenyl] succinamides (**6a, c; 7a, c**) and N-(4-benzylsulfamoyl-phenyl) succinamides (**6b, d; 7b, d**) (Figure 1).

Potato disc is a useful test for monitoring the inhibition of crown-gall tumors (6, 7). Crown gall is a neoplastic disease of plants induced by specific strains of the Gramnegative bacterium *Agrobacterium tumefaciens* (8), first reported by Smith and Townsend (9) and Jensen (10). This malignancy, normally affecting dicotyledonous plants, is induced by inoculation of a wound site with A. tumefaciens followed by the transfer of a large plasmid from *A. tumefaciens* to the plant (11). The genetic information (TDNA) of the large plasmid transforms normal plant cells into autonomous tumor cells (12).

Once the tumor induction has taken place, the autonomous proliferation of the tumor cells becomes entirely independent of the bacteria (13). Galsky (14) showed that inhibition of crown gall tumor initiation on potato discs showed good agreement with compounds and plant extracts known to be active in the 3PS (*in vivo*, mouse leukemia) antitumor assay. In addition to the inhibition of tumor initiation also the inhibition of the growth of the tumors agrees well with 3PS activity (15).

Activity of the compounds **6a-d, 7a-d** was based on the method described above.

## MATERIALS AND METHODS

### Chemical

All the chemicals and reagents were purchased from Merck and Aldrich. 4-acetamidobenzenesulfonyl chloride was synthesized from acetanilide and chlorosulfonic acid according to published procedure. The IR spectra were measured on a Unicam SP-1100 spectrophotometer, using samples prepared as KBr disks. The ^1^H NMR spectra were recorded on a Brucker AC-80 spectrometer (Germany) operating at a working frequency of 80 MHz, using tetramethylsilane as the internal standard. The samples were dissolved in DMSO-d_6_ or CD_3_OD.

**General method for preparation of N-substituted 4-acetamidobenzenesulfonamides (3a, b).** To 4-acetamidobenzenesulfonyl chloride (24 g, 0.1 mol) dissolved in diethyl ether (300 ml) was added dropwise related amine (0.21 mol). The mixture was stirred at room temperature for 1h. The mixture was poured into ice-water. The precipitate was filtered and dried to give 4-acetamido-N-substituted benzenesulfonamides (**3a, b**).

**General method for preparation of 4-amino N-substituted benzenesulfonamides (4a, b).** To 4-acetamido-N-substituted benzenesulfonamides (**3a, b**, 0.08 mol) in water (100 ml) was added concentrated HCl (50 ml). The mixture was heated for 1 h. until a clear solution was obtained. The heating was continued for additional 10 min. The clear solution was cooled to room temperature and neutralized with adding sodium hydroxide (10%) followed by saturated solution of sodium bicarbonate. A precipitate was formed which was filtered and washed with cold water and dried to give 4-amino N-substituted benzenesulfonamides (**4a, b**).

**Methyl 3-chlorocarbonylpropionate (5)**. Succinic anhydride (10g, 0.1 mol) was refluxed in methanol (40 ml) for 8h. The excess methanol was evaporated in vacuum. An oily residue, succinic acid monomethyl ester (13.12 g) was obtained. Thionyl chloride (15 ml) was added dropwise to the residue during 15 min, while stirring. The mixture was then refluxed for 5 h. Excess thionyl chloride was evaporated in vacuum. The greenish yellow liquid, methyl 3-chlorocarbonylpropionate was used directly in next step without any further purification.

**N-substituted succinamic esters (6a, b).** Compound **5** (4 ml, 0.02 mol) was added dropwise to the stirring solution of compound **4** (0.02 mol) in dry tetrahydrofuran (30 ml) and pyridine (4 ml). Stirring was continued overnight and the solution was evaporated to near dryness in vacuum. Water (20 ml) and a few drops of concentrated HCl were added to the residue to acidify it. The precipitate was filtered and washed with saturated solution of sodium bicarbonate (50 ml) and twice with distilled water (50 ml) to give N-substituted succinamic esters (**6a, b**).

**N-substituted succinamic acids (6c, d).** To a stirring solution of compound **4** (7.6 mmol) in dry acetone (20 ml), succinic anhydride (0.8 g, 8 mmol) was added and refluxed for 2 h. Acetone was removed with Rota-evaporator. The residue was basified with saturated solution of sodium bicarbonate (10 ml), stirred for 5 min and filtered. The filtrate was acidified with concentrated HCl to pH=2. The precipitate was filtered and then washed twice with distilled water (50 ml). The precipitate was dried to give N-substituted succinamic acids (**6c, d**).

**General method for preparation of N, N’-disubstituted succinamides (7a-d).** Compounds (**6a-d**) (10 mmol), ammonium chloride (0.2 g) and suitable amine (10 mmol) were dissolved in tetrahydrofuran (15 ml). The mixture was refluxed for 8 h. The progress of reaction was monitored by thin layer chromatography (TLC). The solvent was removed and the residue was washed with HCl (0.1 N, 50 ml) and cold distilled water (50 ml) to give after dryness N, N’-disubstituted succinamides (**7a-d**).

### Screening with the potato disc assay

Fresh, disease-free potatoes were obtained from a local market. Tubers of moderate size were surface sterilized by immersion in sodium hypochlorite 0.1% for 20 min. Ends were removed and the potatoes were soaked for an additional 10 min in sodium hypochlorite solution. A core of the tissue was extracted from each tuber with a surface-sterilized 1.0 cm cork borer. Pieces of 2 cm were removed from each end and discarded. The remainder of the cylinder was cut into 0.5 cm discs with a surface-sterilized scalpel. The discs were then transferred to agar plates (1.5 g of agar dissolved in 100 mL double distilled water (DDW), autoclaved for 20 min at 121°C, 20 mL poured into each Petri dish). Each plate contained 5 discs and 3-5 plates, were used for each sample dilution.

*A. tumefaciens* (ATCC 23341) was cultivated in Soybean Casein Digest Agar. For inoculation of the potato discs, 48 h broth culture containing 5 × 10^9^ cells/ml was used. Samples were dissolved in 5% DMSO, filter sterilized, diluted and mixed with the bacterial culture for inoculation.

The potato discs were incubated for 20 days at 25°C incubator, after which Lugol’s solution (I_2_/KI) was added, the tumor counts were made and compared with negative controls (bacterial suspension containing 5% DMSO). The results were expressed as + or - percentage versus the number of tumors on the control discs. Significant activity was indicated by consistent negative values of ca. 20% or greater inhibition. Vincristine was used as positive control.

## RESULTS AND DISCUSSION

### Chemistry

**Figure 2:**
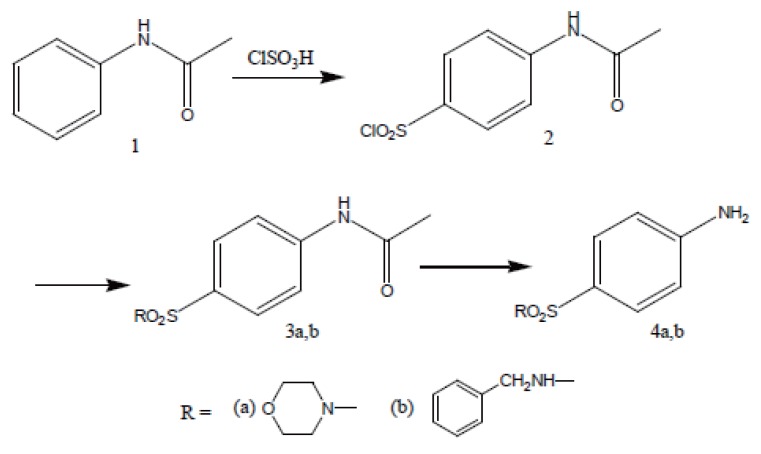
Synthesis of intermediates **4a, b**.

Acetanilide (**1**) was converted to its corresponding sulfonyl chloride (**2**). Condensation of **2** with related amines (morpholine and benzylamine) gave 4-acetamido-N-substituted benzenesulfonamides (**3a, b**). Removal of protecting acetyl group afforded N-substituted benzenesulfonamides (**4a, b**) (Figure 2).

**Figure 3:**
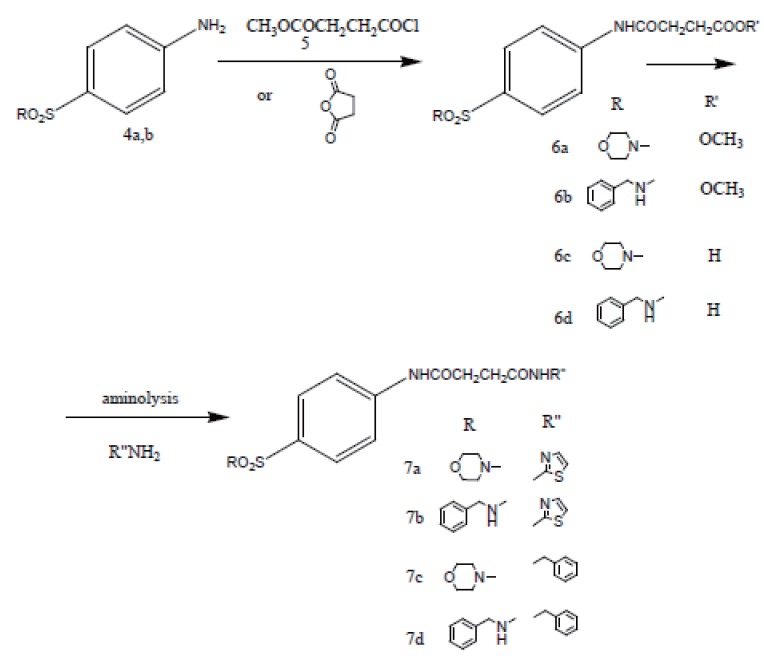
Synthesis of final succinamides **6a-d** and **7a-d**.

Condensation of the N-substituted benzenesulfonamides (**4a, b**) with succinic anhydride or methyl 3-chlorocarbonylpropionate (**5**), prepared from succinic anhydride, afforded N-substituted succinamic esters (**6a, b**) and acids (**5c, d**). Aminolysis of (**6a, b**) with corresponding amines (2-aminothizole and benzylamine) gave the succinamides (**7a-d**) as shown in Figure 3.

The synthesized compounds appeared as white crystalline substances stable at room temperature. The compounds were insoluble in water and soluble in DMSO.

The proposed structure of compounds was confirmed by IR and ^1^H NMR spectroscopy. Interpretation of the ^1^H NMR spectra was base on the chemical shifts, multiplicities, and integral intensities of the signals. The yields and physicochemical properties of compounds **3-7** are presented in Table 1.

**Table 1:** Physicochemical characteristics of compounds **3-7**

Compound	Yield %	M.P. °C (methanol)	Empirical formula	IR spectrum: ν_max_, cm^-1^		1H NMR (DMSO-d_6_) spectrum: δ, ppm
				CO	NH	

3a	85	103-105	C_12_H_16_N_2_O_4_S	1690	3350, 3450	10.4(s, 1H, NH), 7.93(m, 4H, arom), 3.73(m, 4H, CH_2_), 2.9(m, 4H, CH_2_), 2.1(s, 3H, CH_3_)
3b	89	129-131	C_15_H_16_N_2_O_3_S	1680	3300, 3150	10.4(s, 1H, NHCO), 7.9(t, 1H, NHSO_2_), 7.8(s, 4H, arom), 7.3(s, 5H, arom), 4(d, 2H, CH_2_), 2.1(s, 3H, CH_3_)
4a	93	210-212	C_10_H_14_N_2_O_3_S	1640	3375, 3275	7.9(d, 2H, arom), 7.2(d, 2H, arom), 6.1(s, 2H, NH_2_), 3.8(m, 4H, CH_2_), 2.9(m, 4H, CH_2_)
4b	89	159-161	C_13_H_14_N_2_O_2_S	1650	3350, 3450, 3270	7.4-6.7(m, 12H, arom, NH), 4(s, 2H, CH_2_)
6a	91	138-140	C_15_H_20_N_2_O_6_S	1720	3350, 3250	10.4(s, 1H, aryl-NHCO), 7.7(m, 4H, arom), 3.8(m, 7H, CH_2_-morpoline, OCH_3_), 2.9(m, 4H, CH_2_-morpholine), 2.6(s, 4H, CH_2_)
6b	95	150-152	C_18_H_20_N_2_O_5_S	1725	3350, 3250	10.4(s, 1H, aryl-NHCO), 8(t, 1H, NHSO_2_), 7.6(s, 4H, arom), 7.3(s, 5H, arom), 4(d, 2H, CH_2_NHSO_2_), 3.6(s, 3H, CH_3_O), 2.6(s, 4H, CH_2_-succinic)
6c	88	220-221	C_14_H_18_N_2_O_6_S	1640	3275, 3250	10.4(s, 1H, aryl-NHCO), 7.9-7.4(m, 4H, arom), 4-1.9(m, 12H, CH_2_)
6d	92	169-170	C_17_H_18_N_2_O_5_S	1640	3260, 3250	10.4(s, 1H, aryl-NHCO), 8(t, 1H, NHSO_2_), 7.6(s, 4H, arom), 7.3(s, 5H, arom), 4(d, 2H, CH_2_NHSO_2_), 2.6(s, 4H, CH_2_-succinic)
7a	85	160-165	C_17_H_20_N_4_O_5_S_2_	1690	3370, 3330	9.8(m, 2H, aryl-NHCO), 7.3-6.8(m, 6H, arom, H_4,5_-thiadiazole), 3.3-2.6(m, 4H, CH_2_-morpholine)2.3-1.8(m, 8H, CH_2_-morpholine, CH_2_-succinic)
7b	90	170-173	C_20_H_20_N_4_O_4_S_2_	1680	3365, 3300	9.8(m, 2H, aryl-NHCO), 7.3(t, 1H, NHSO_2_), 7.03-6.3(s, 6H, arom, H_4,5_-thiadiazole), 3.3 (d, 2H, CH_2_NHCO), 2.8(m, 4H, CH_2_-succinic)
7c	86	155-157	C_21_H_25_N_3_O_4_S	1700	3370, 3330	10.5(s, 1H, aryl-NHCO), 8.5(m, 2H, NHCO), 7.8(m, 4H, arom), 7.3(s, 5H, arom), 4.3(d, 2H, CH_2_NHCO), 3.6(4H, CH_2_-morpholine), 2.9(m, 4H, CH_2_-morpholine), 2.5(m, 4H, CH_2_-succinic)
7d	84	170-172	C_24_H_25_N_3_O_4_S	1650	3290	10.4(s, 1H, aryl-NHCO), 8.5(t, 1H, NHCO), 8(bs, 1H, NHSO_2_), 7.8(s, 4H, arom), 7.3(s, 5H, arom), 4.3(d, 2H, CH_2_NHCO), 4(bs, 2H, CH_2_NHSO_2_), 2.5(m, 4H, CH_2_-suc-cinic)

### Biological Assay

The antitumor activity of compounds was studied using the potato disc bioassay technique. Minimum inhibitory concentration (MIC) of samples on *A. tumefaciens* was found to be greater than 1 mg/ml. The formation of formazan pink color developed after addition of MTT dye did not give rise to color at lower concentrations of the tested compounds indicating no inhibition for *A. tumefaciens* growth. The concentrations above the MIC would produce false positive results in potato disc assay due to the antibacterial activity of the compounds and preventing the bacterium to induce the tumogenesis. Therefore, dilutions below MIC including 0.01, 0.1 and 1 mg/ml were used for potato disc assay. The results have been shown in Table 2. Among these N-Benzyl-N'-(4-benzylsulfamoyl-phenyl)-succinamide (**7c**) and N-Benzyl-N’-[4-(morpholin-4-ylsulfonyl)-phenyl]-succinamide (**7d**) exhibited significant inhibition of crown gall tumors caused by *Agrobacterium tumefaciens* at 10 microgram/ml concentration. The activity of compounds **6a, 7b** and **7d** was comparable to that of vincristine. The results have been shown in Table 2. Vincristine at 0.25 mg/ml was employed as positive control and caused -67.24% inhibitions. Compound **7b** at 1 mg/ml caused -80.50% tumor inhibitions with highest activity among compounds tested.

**Table 2:** Antitumor activity of compounds 6a-d, 7a-d on potato disc model^a^

Compound	R_1_	R_2_	Concentration (mg/ml)	Percent growth (± SEM)

6a	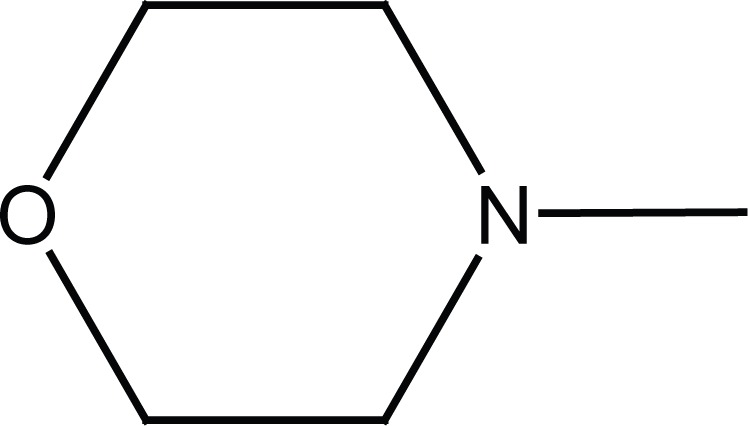	OCH_3_	1	-47.5(±7.8)
			0.1	-63.17(±4.3)
			0.01	-19.76(±4.6)
6b	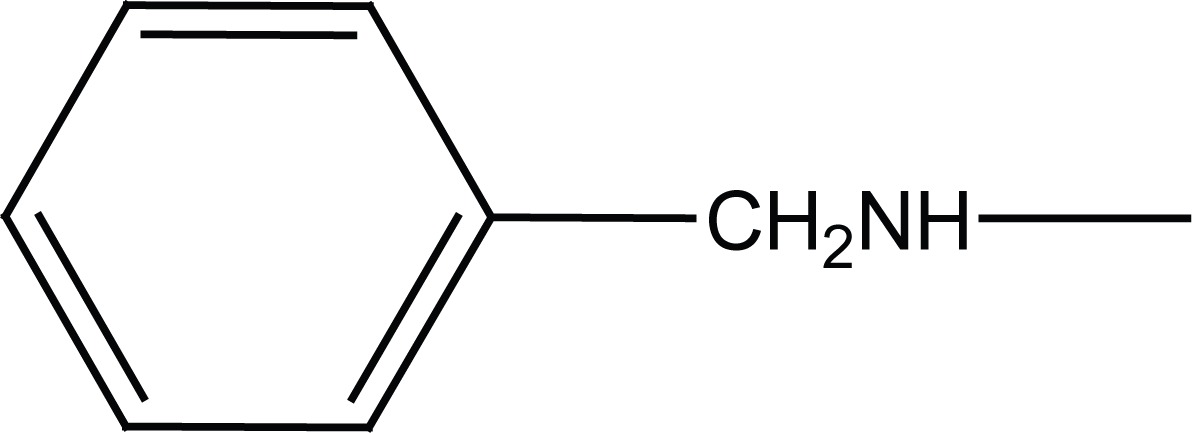	OCH_3_	1	-41.54(±4.3)
			0.1	-17.17(±7.6)
			0.01	-19.24(±4.5)
6c	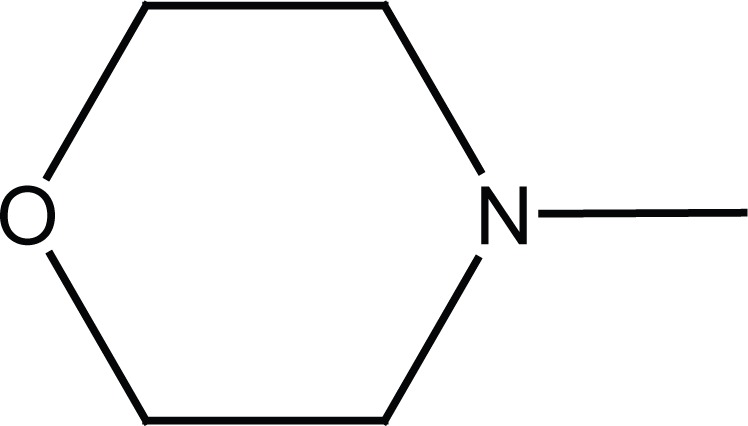	OH	1	-46.30(±4.5)
			0.1	-39.00(±3.7)
			0.01	-22.81(±2.4)
6d^a^	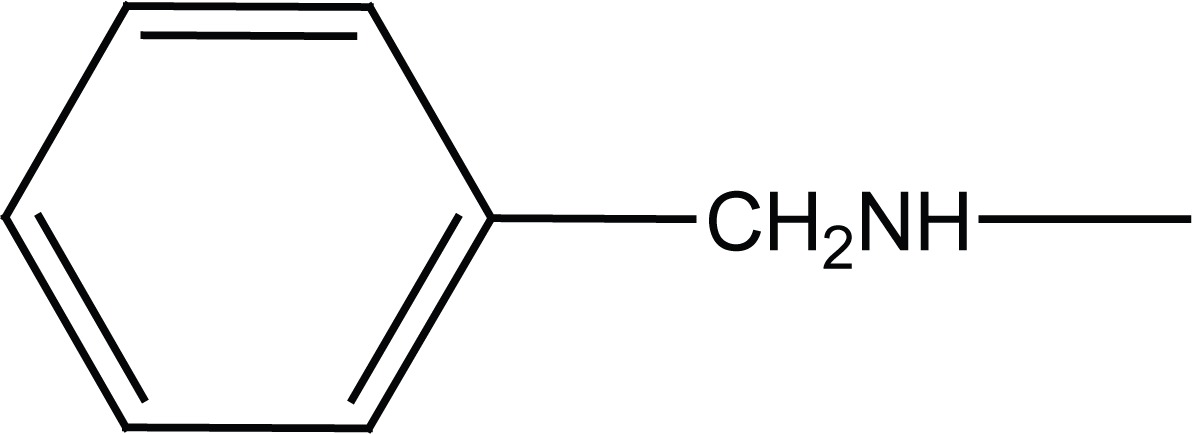	OH	1	nd^b^
			0.1	nd
			0.01	nd
7a	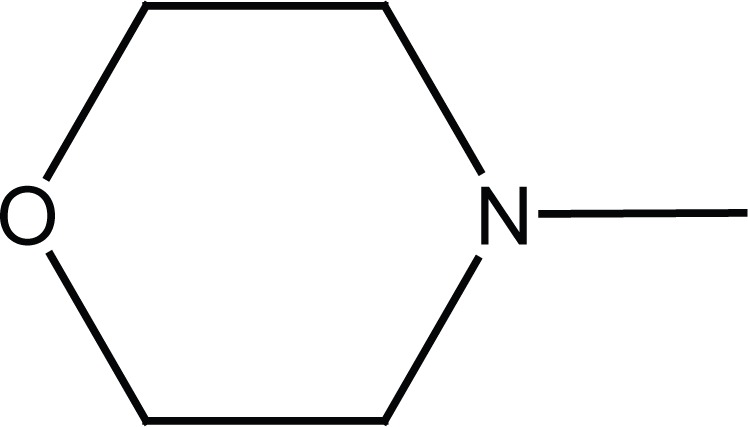	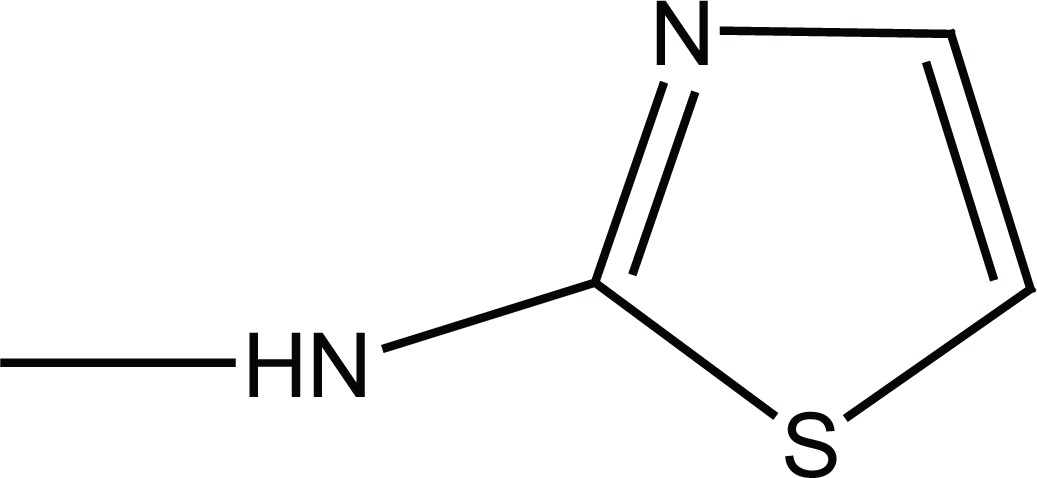	1	-28.37(±5.2)
			0.1	-27.76(±9.4)
			0.01	-19.72(±9.9)
7b	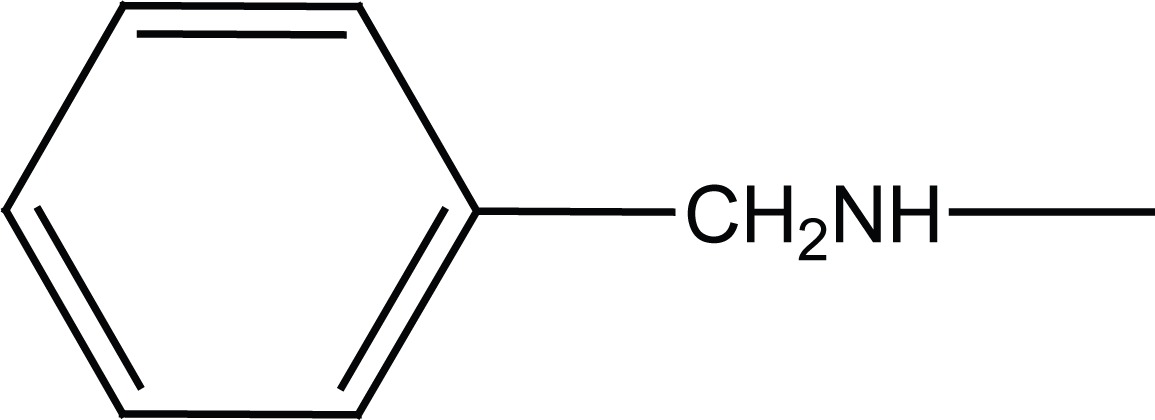	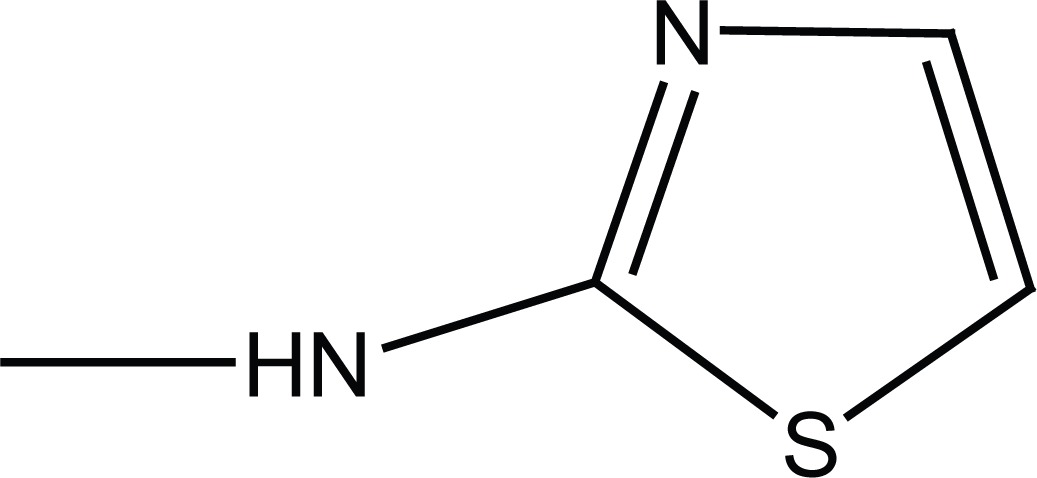	1	-80.5(±9.3)
			0.1	-52.04(±5.1)
			0.01	-19.24(±8.7)
7c	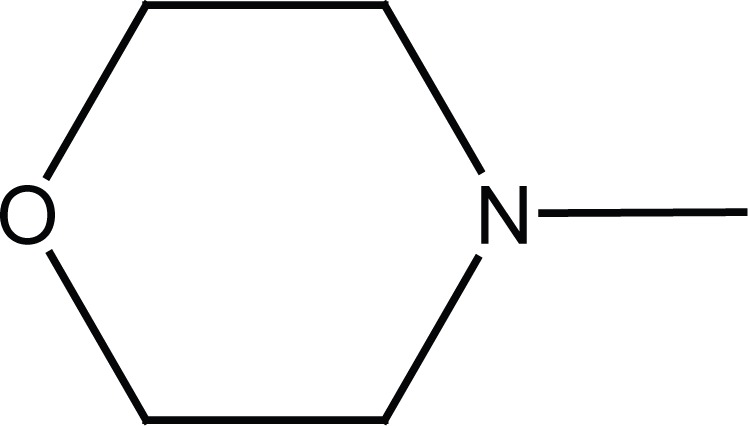	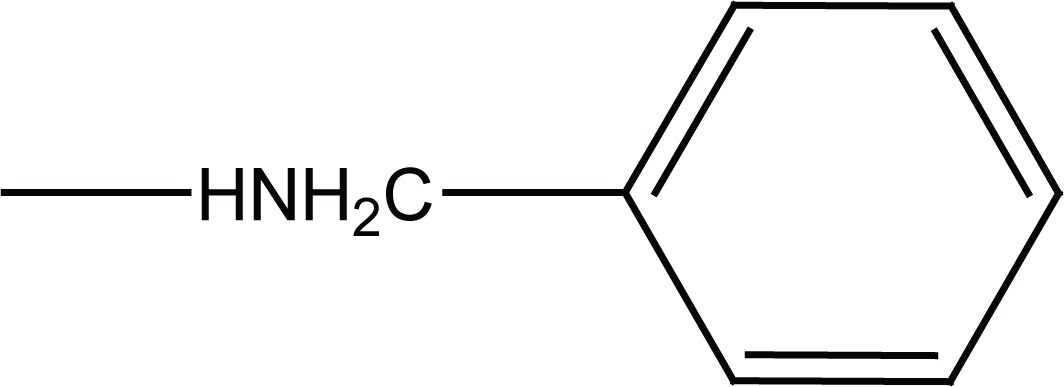	1	-39.13(±6.8)
			0.1	-37.62(±2.6)
			0.01	-35.09(±4.9)
7d	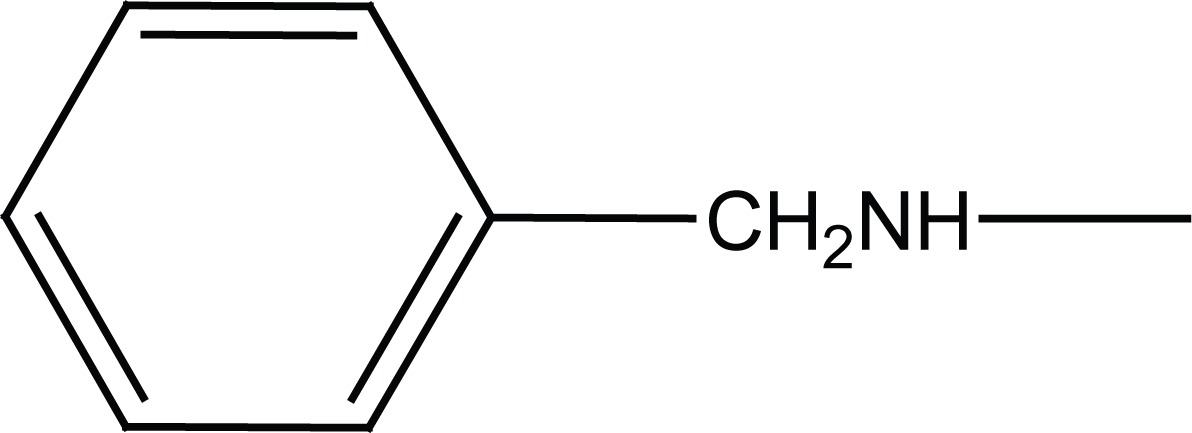	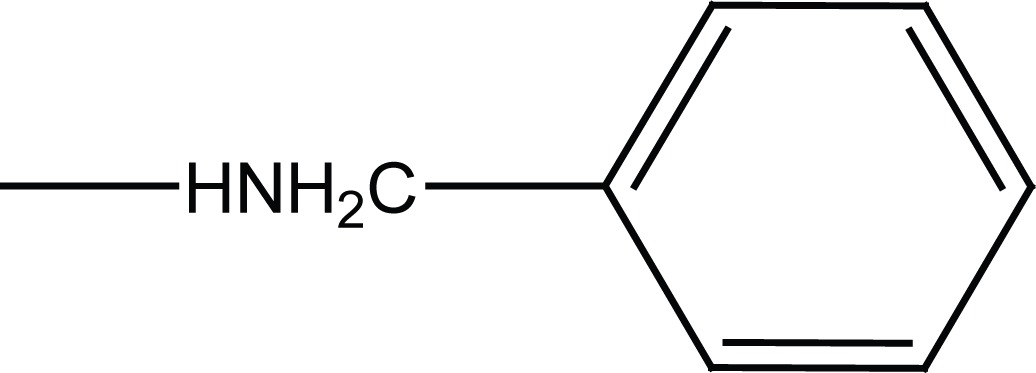	1	-57.44(±2.8)
			0.1	-56.17(±6.5)
			0.01	-18.84(±1.5)
vincristine			0.25	-67.24 (±3.8)

a
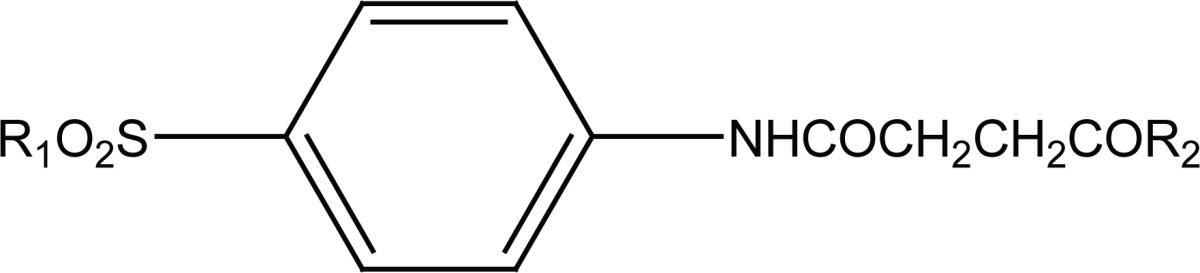
;

b not determined.
